# The Real-World Impact of PARP Inhibitor Maintenance Therapy in High Grade Serous Tubo-Ovarian and Peritoneal Cancers

**DOI:** 10.3390/cancers17213591

**Published:** 2025-11-06

**Authors:** Maryam Al-Ani, Bahaaeldin Baraka, Navin Mathiyalagan, Muhammad Adeel Sarwar, Avinash Segaran, Wafaa Abuzahra, Alayna Radford, Kersty Buxton, Lalith Seneviratne, Santhanam Sundar, Anjana Anand, David Nunns, Karin Williamson, Ben Wormald, Ketankumar Gajjar, Srinivasan Madhusudan

**Affiliations:** 1 Department of Oncology, Nottingham University Hospitals, Hucknall Road, Nottingham NG5 1PB, UKn.mathiyalagan@nhs.net (N.M.);; 2 Department of Gynaecology, Nottingham University Hospitals, Hucknall Road, Nottingham NG5 1PB, UK; 3 Naaz-Coker Ovarian Cancer Research Centre, Biodiscovery Institute, School of Medicine, University of Nottingham, University Park, Nottingham NG2 7RD, UK

**Keywords:** ovarian cancer, high grade serous tubo-ovarian cancer, PARP inhibitors, real world evidence

## Abstract

Background: Pivotal clinical trials have led to the routine clinical use of PARP inhibitor (PARPi) (olaparib, niraparib, or rucaparib) maintenance therapy in high-grade serous tubo-ovarian and peritoneal cancers. Whether various PARPis have comparable clinical impact in the real-world is an area of ongoing investigation. Methods: We conducted a retrospective study of all patients who received PARPi maintenance therapy at Nottingham Cancer Centre from October 2017 to April 2025. Clinical data were extracted from multidisciplinary team electronic health records, including age, BRCA mutation status, HRD status, treatment history, type of PARP inhibitor received, progression-free survival (PFS), and overall survival (OS). Results: A total of 177 patients had received PARPi therapy with a mean age of 63 years at diagnosis. In all, 94/177 (53.1%) had received PARPi as primary maintenance, while 83/177 (46.9%) were treated in the recurrent setting. All together, 25/177 (14.1%) had BRCA1 germline mutation and 21/177 (11.9%) had BRCA2 germline mutation. In the primary olaparib setting, PFS was significantly better in BRCA2 germline-mutated patients compared to BRCA1 germline-mutated patients [median PFS was not reached vs. 29.0 months, respectively, *p* = 0.002]. In BRCA, wild-type patients receiving primary niraparib, median PFS was 11 months. Median PFS for patients with upfront surgery was 37 months compared to 19 months in the interval debulking group but not significant (*p* = 0.49). In the recurrent setting, there was no significant difference in median PFS between niraparib and rucaparib [10 months vs. 9 months, *p* = 0.594]. Conclusions: BRCA2 germline-mutated patients obtained significantly greater benefit from olaparib compared to BRCA1-mutated patients. PFS benefit from niraparib (primary or recurrent setting) is comparable to clinical trials. There was no difference in benefit between niraparib and rucaparib in the recurrent setting.

## Simple Summary

PARP inhibitor (olaparib, niraparib, or rucaparib) maintenance treatment is a standard treatment option in patients with high-grade serous tubo-ovarian and peritoneal cancers following surgery and chemotherapy. Whether olaparib, niraparib, or rucaparib have similar clinical benefit is an area of clinical investigation. The real-world data evaluation of olaparib, niraparib, and rucaparib maintenance can provide valuable insight that can aid clinical decision-making. We conducted a retrospective observational study at our centre to evaluate survival outcomes and compare clinical trial data. We observed significantly better survival benefit from olaparib in BRCA2 germline mutated patients compared to BRCA1 mutated patients. In the recurrent setting, niraparib and rucaparib provide similar clinical benefits.

## 1. Introduction

Ovarian cancer ranks as the 6th most common cancer affecting females in the UK [1]. Ovarian cancer causes approximately 4100 deaths per year in the UK [1]. high-grade serous ovarian cancer (HGSOC) is the most common subtype of epithelial ovarian cancer (EOC), which accounts for over 70% of all ovarian cancer types [2]. While early disease has a high chance of cure, most patients present with late-stage disease and 75% of these patients will have poor outcomes from their disease [3]. For decades, the cornerstone of therapy was cytoreductive surgery with a combination of platinum–taxane–based chemotherapy [4]. The BRCA1 and BRCA2 genes were discovered in the early 1990s, which revolutionized risk assessment and genetic counselling for families affected by ovarian and breast cancer [4]. Inheritance of BRCA 1 and BRCA 2 mutations confers approximately 40% and 20% lifetime risk of developing ovarian cancer, respectively [5]. In addition to surgery and combination chemotherapy, the use of targeted therapy, namely, poly-ADP ribose polymerase (PARP) inhibitors in HGSOC management led to a paradigm shift in survival outcomes during the recent decade, particularly in patients with BRCA mutations or homologous recombination deficiency or platinum-sensitive HGSOC [6,7]. The mechanism of action of PARP inhibitors is complex. At the NAD^+^ binding site, PARP 1/2 catalytic activity is competitively inhibited [8]. Single-strand break repair, a pathway related to base excision repair (BER) is hampered by PARP inhibition, leading onto accumulation of single-strand DNA breaks (SSBs). During replication, these unresolved SSBs lead to replication fork collapse and the formation of double-strand breaks (DSBs). During the G1-S phase of the cell cycle, DSBs are processed through homologous recombination (HR). In HR deficient cells, such as those that lack BRCA1 or BRCA2, DSBs accumulate leading to synthetic lethality [6,7]. In addition, the stabilization of PARP-DNA complexes, a phenomenon known as “PARP trapping” leads to replication stalling, and if unrepaired leads to DSB accumulation, promoting cell death [6,7]. The ability of PARP inhibitors to trap PARP varies with Talzoparib exhibiting the highest PARP trapping efficiency compared to nirapririb, rucaparib, and olaparib [9].

The pharmacokinetic and pharmacodynamic distinctions among PARP inhibitors significantly affect their clinical application, including dosing schedules, interaction potential, and toxicity profiles. Niraparib is administered once daily (half-life: 36–51 h), whereas olaparib (half-life: ~12 h) and rucaparib (half-life: ~26 h) require twice-daily dosing [10]. These agents are also metabolized through distinct pathways. Niraparib undergoes hydrolysis by carboxylesterases, reducing dependence on hepatic cytochrome P450 (CYP) enzymes and limiting drug–drug interaction risk; in contrast, olaparib is primarily metabolized by CYP3A4, and rucaparib by CYP1A2, CYP2D6, and CYP3A4, increasing susceptibility to metabolic interactions [11].

Toxicity profiles further reflect mechanistic differences. Niraparib demonstrates higher rates of grade 3/4 thrombocytopenia (34%), potentially due to off-target inhibition of dopamine and norepinephrine transporters, which may also contribute to hypertension [12]. Rucaparib commonly elevates serum creatinine (92%) via inhibition of renal MATE/OCT transporters—without true nephrotoxicity—and may also cause transient transaminitis (grade 3/4: 12%) [11]. Anemia is frequently observed with both olaparib and rucaparib [12]. Collectively, these agent-specific pharmacological and mechanistic properties—spanning trapping efficiency, pharmacokinetics, metabolism, and toxicity—underscore the importance of biomarker-guided, individualized therapy selection. Priorities for toxicity management, concurrent medications, patient comorbidities, and tumour HRD status must all be incorporated into such an approach [13].

Olaparib received National Institute of Clinical Excellence (NICE) approval in 2019 for patients with FIGO stage III/IV BRCA-mutated ovarian cancer in the maintenance setting following response to first-line platinum-based chemotherapy or following the first or subsequent relapse where they responded to re-challenge with platinum-based chemotherapy provided the patient has not previously received a PARP inhibitor [14]. Niraparib is funded by the NHS in England for patients with or without BRCA mutations. It can be used as a first-line maintenance treatment for those with high-grade stage III or IV ovarian, fallopian tube, or primary peritoneal cancer who have responded to platinum-based chemotherapy regardless of BRCA status, though separate approval forms apply depending on whether the patient has a BRCA mutation [15]. While niraparib was also briefly approved for use after the first relapse, that indication was withdrawn in 2022. As for rucaparib, it was first approved in 2019 for second-line maintenance, and since 2025, it has been available as a first-line option for BRCA–wild-type patients, regardless of their HRD status, as long as they have responded to platinum-based treatment [16].

The real-world data evaluation of olaparib, niraparib, and rucaparib maintenance for HGSOCs in the UK is an area of ongoing investigation. We conducted a retrospective observational study at our centre to evaluate survival outcomes and to compare these with clinical trial data.

## 2. Materials and Methods

### 2.1. Study Design

A retrospective observational study was conducted at the Nottingham Cancer Centre, Nottingham University Hospitals NHS Trust, to assess outcomes in patients with high-grade serous epithelial ovarian, fallopian tube, or primary peritoneal cancer treated with PARP inhibitors (olaparib, niraparib, or rucaparib) between October 2017 and April 2025. The study received approval from the Clinical Effectiveness Team (Reference: 24-774C, approval date: 12 December 2024). The study was considered an audit under NHS research governance. All data were anonymized, and patient confidentiality was maintained in accordance with institutional data protection and research governance guidelines. Individual patient consent was not required for this retrospective audit.

### 2.2. Patients

Patients aged ≥ 18 years with histologically confirmed high-grade serous epithelial ovarian, fallopian tube, or primary peritoneal carcinoma were eligible for inclusion. Patients must have received a PARP inhibitor as maintenance therapy following chemotherapy, either in the primary or recurrent setting. Eligibility was not restricted by BRCA or HRD status. Patients were excluded if they had a non-ovarian primary cancer, insufficient clinical documentation, or discontinued treatment early due to non-disease-related reasons.

As per national guidelines, all patients with HGSOCs were tested for pathogenic variants in *BRCA1*, *BRCA2*, *PALB2*, *MLH1*, *MSH2*, *MSH6*, *BRIP1*, *RAD51C,* and *RAD51D* by DNA sequencing at the Cambridge Genomic Laboratory Hub (R207, https://www.england.nhs.uk/publication/national-genomic-test-directories/, accessed on 1 April 2025). HRD status was assessed by HRD genomic instability testing in tumour samples using Illumina TSO500 HRD assay.

### 2.3. Data Collection

Clinical data were extracted from multidisciplinary team electronic health records, including age, BRCA mutation status, HRD status, treatment history, and type of PARP inhibitor received. Out of 182 patients initially screened, 177 met all eligibility criteria and were included in the final analysis.

### 2.4. Statistical Analysis

Descriptive statistics were used to summarize patient demographics and clinical characteristics. Median overall survival (mOS) and median progression-free survival (mPFS) were calculated using the Kaplan–Meier method. Group comparisons—for example, between BRCA1 and BRCA2 status, or type of PARP inhibitor—were assessed using the log-rank (Mantel–Cox) test. A *p*-value of ≤ 0.05 was considered statistically significant. All analyses were conducted using IBM SPSS Statistics, version 29.0.1.0.

## 3. Results

**Figure 1:**
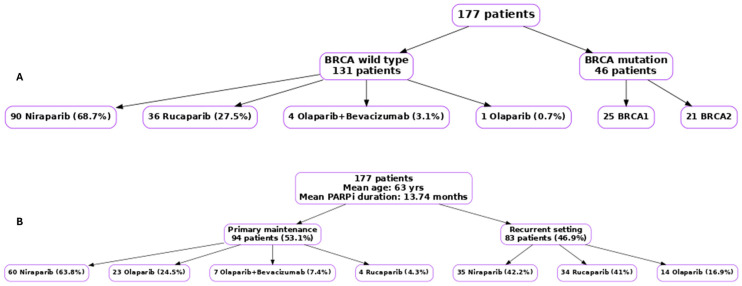
Flowcharts summarizing patient characteristics and PARPi treatment. (**A**) BRCA status (wild-type vs. mutated) and (**B**) treatment setting (primary vs. recurrent).

Patient clinical characteristics and PARPi treatment, BRCA status and treatment settings are summarized in Table 1. The mean age at diagnosis was 63 years. The mean duration of PARPi maintenance treatment was 13.74 months. The median follow-up time was 28 months. Treatment characteristics are summarized in Figure 1. A total of 94/177 (53.1%) received PARPi as primary maintenance while 83/177 (46.9%) were treated in the recurrent setting. In the primary setting, the most used agent was niraparib (60/94, 63.8%) followed by olaparib (23/94, 24.5%), olaparib + bevacizumab (7/94, 7.4%), and rucaparib (4/94, 4.3%). For patients treated with PARPi in the disease recurrence setting, comparable use of niraparib and rucaparib were noted with 35/83 (42.2%) and 34/83 (41%), respectively. Olaparib was used in 14/83 (16.9%) of patients. Out of the 177, 131 patients had BRCA–wild-type of whom 90 patients (68.7%) received niraparib, 36/131 (27.5%) had rucaparib, 4 patients (3.1%) received olaparib/bevacizumab, and only one patient had olaparib maintenance therapy (Figure 1).

### 3.1. Survival Outcomes in Patients with Germline BRCA Mutations Receiving Olaparib as Primary Maintenance Therapy

**Figure 2:**
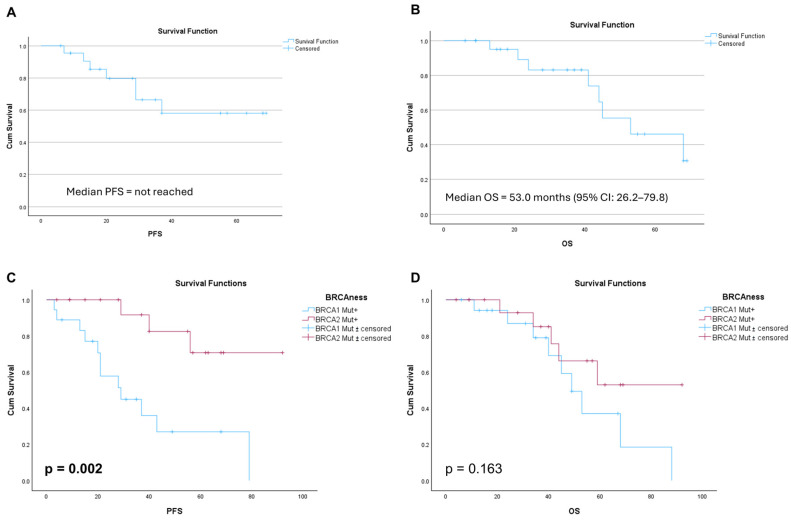
Survival outcomes in the BRCA cohort. Kaplan–Meier curves showing progression-free survival (**A**) and overall survival (**B**) in the BRCA-mutated cohort receiving olaparib as primary maintenance therapy. Kaplan–Meier curves comparing BRCA1 vs. BRCA2 subgroups: (**C**) progression-free survival (PFS) and (**D**) overall survival (OS) in patients receiving olaparib as primary maintenance therapy.

For the whole BRCA-mutated cohort receiving olaparib as maintenance therapy in the primary setting, median PFS was not reached, reflecting sustained disease control (Figure 2A). The mean PFS was 49.9 months (95% CI: 38.7–61.1). Overall survival analysis showed that the median OS was 53.0 months (95% CI: 26.2–79.8) (Figure 2B). Mean OS was 51.9 months (95% CI: 42.6–61.3). We then evaluated individual subgroups. BRCA1-mutated patients had a median PFS of 29.0 months (95% CI: 13.8–44.2) and a mean PFS of 38.2 months (95% CI: 23.8–52.5). In BRCA2-mutated patients, the median PFS was not reached, indicating prolonged disease control while the mean PFS was 77.7 months (95% CI: 63.9–91.6). The difference in PFS between BRCA1 and BRCA2 subgroups was statistically significant (log-rank [Mantel–Cox] test: *p* = 0.002; Figure 2C).

BRCA1-mutated patients had a median OS of 49.0 months (95% CI: 38.3–59.7) and a mean OS of 53.7 months (95% CI: 39.8–67.6). The median OS for BRCA-2 patients was not reached, implying sustained survival. Meanwhile, these patients had a longer mean OS of 68.7 months (95%CI: 53.1–84.3). No statistically significant difference in OS was found between BRCA1 and BRCA2 subgroups (log-rank [Mantel–Cox] test: *p* = 0.163; Figure 2D).

### 3.2. Survival Outcomes for Patients Who Received Olaparib as Maintenance Therapy in the Recurrent Disease Setting

The median PFS for patients treated with olaparib was 43.0 months (95% CI: 15.5–70.5), compared with 10.0 months for niraparib and 9.0 months for rucaparib. Due to the small number of olaparib-treated patients, 14 patients, no formal statistical testing was performed.

### 3.3. Survival Outcomes in BRCA Wild Type Receiving Niraparib Primary Maintenance

Median PFS in patients who received niraparib in the primary maintenance setting was 11 months (95% CI: 7.12–14.88). Median OS was 29 months (95% CI: 24.89–33.11).

### 3.4. Survival Outcomes in Patients Who Received Primary Surgery or Neoadjuvant Chemotherapy Followed by Surgery

**Figure 3:**
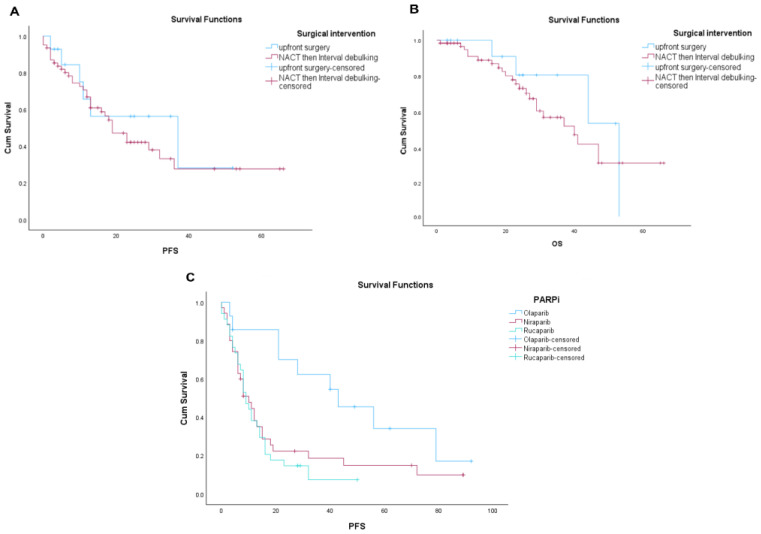
Kaplan–Meier curves comparing (**A**) progression-free survival (PFS) and (**B**) overall survival (OS) between patients undergoing upfront surgery versus interval debulking after neoadjuvant chemotherapy. (**C**) Kaplan–Meier curve of progression-free survival (PFS) comparing patients treated with olaparib, niraparib, and rucaparib.

A subset of 80 patients from the full cohort were suitable for this analysis (4 patients were deemed unfit for surgery, 8 patients had declined surgery, and 2 patients had previous prophylactic bilateral salpingo-oophorectomy). Out of the 80 patients, 14/80 had upfront surgery and 62/80 had interval debulking surgery post neoadjuvant chemotherapy. In all, 4/80 were inoperable after neoadjuvant chemotherapy. All patients received PARPi maintenance therapy. Altogether, 66/80 patients had PARPi as primary maintenance and 14/66 had PARPi as maintenance following disease recurrence. Median PFS for patients with upfront surgery was 37 months (95% CI: 1.481 to 72.50), while median PFS for patients who had interval debulking post neoadjuvant chemotherapy was 19 months but did not reach statistical significance (*p* = 0.49) (Figure 3A). Median overall survival for patients who had upfront surgery was 53 months versus 40 months for patients who had interval debulking; this difference was not statistically significant (*p* = 0.50) (Figure 3B).

### 3.5. Survival Outcomes for Olaparib, Niraparib, and Rucaparib in the Recurrent Setting

The median PFS was 43.0 months (95% CI: 15.5–70.5) for olaparib, 10.0 months (95% CI: 5.6–14.4) for niraparib, and 9.0 months (95% CI: 6.6–11.4) for rucaparib. The overall difference was statistically significant (log-rank *p* = 0.012) (Figure 3C). However, the data should be interpreted with caution given the fewer number of patients receiving olaparib in this cohort. No significant difference in PFS was observed between niraparib and rucaparib (*p* = 0.594) (Figure 3C).

### 3.6. Survival Outcomes in HR-Deficient (HRD) or HR-Proficient (HRP) Tumours

HRD status evaluation was introduced recently for HGSOC patients through the Genomic Medicine Service Alliance (GMSA) in the NHS. HRD status was not available for 138/177 patients (78%). Of those tested, 20/177 (11.3%) tumours were HRD and 19/177 (10.7%) were HRP. The median follow-up for HR-proficient patients was 21.7 months, and the median follow up for HR-deficient patients is 17.8 months.

Because outcomes for primary and recurrent disease are not directly comparable, survival analyses were stratified by treatment setting. In the primary cohort, HRD tumours (n = 20) showed a median PFS of 21.0 months (95% CI: 9.9–32.1) and a mean PFS of 26.5 months (95% CI: 15.6–37.4). For HRP tumours (n = 19), the median was not reached due to censoring. They had a mean PFS of 20.4 months (95% CI: 14.5–26.4). Although HRD patients demonstrated numerically longer PFS, the difference did not reach statistical significance (log-rank *p* = 0.707). In the recurrent cohort, only one tumour was HRD, and two tumours were HRP tumours. Therefore, given the very small sample size, statistical comparisons were not performed.

Taken together, these findings indicate that HRD patients in the primary setting demonstrated numerically longer PFS compared with HRP, although the difference was not statistically significant, and the very small recurrent subgroup precludes firm conclusions. Summary of survival outcomes for the aforementioned subgroups is provided in Table 2.

### 3.7. Multivariate Analysis

In the BRCA-mutated cohort receiving olaparib as primary maintenance, multivariate Cox regression, adjusting for FIGO stage, age group, and BMI showed that BRCA2 mutation remained independently associated with longer progression-free survival compared with BRCA1 mutation (adjusted HR = 0.041, 95% CI: 0.003–0.551, *p* = 0.016). Stage (HR = 7.19, *p* = 0.13) and BMI (HR = 0.12, *p* = 0.11) were not statistically significant. Age group showed no meaningful effect, likely due to the small number of patients aged ≥ 70 years. The overall model was statistically significant (χ^2^ = 16.1, df = 4, *p* = 0.003), indicating that the covariates collectively explained variation in PFS.

For overall survival, in the BRCA-mutated cohort receiving olaparib as primary maintenance, multivariable Cox regression for overall survival, adjusting for FIGO stage, BMI, and age group showed no statistically significant independent predictors of OS (overall model *p* = 0.21). BRCA2 mutation was associated with numerically longer survival compared with BRCA1 (adjusted HR = 0.32, 95% CI: 0.05–1.88, *p* = 0.21), consistent with the direction of effect seen for PFS. Higher BMI (≥25 kg/m^2^) showed a borderline association with improved OS (adjusted HR = 0.11, 95% CI: 0.01–1.04, *p* = 0.054). Stage IV disease (vs. stage III) was associated with a higher hazard of death (HR = 6.22, *p* = 0.10). Due to the limited number of deaths, none reached statistical significance.

## 4. Discussion

In this retrospective study, we analyzed 177 patients with high-grade serous tubo-ovarian and peritoneal cancers who received PARP inhibitors (olaparib, niraparib, or rucaparib) as maintenance therapy between 2017 and 2025 at a single large cancer centre.

For the entire BRCA-mutated cohort receiving olaparib as maintenance therapy in the primary setting, the mean PFS was 49.9 months (95% CI: 38.7–61.1), while the median PFS was not reached at the time of data cut-off, indicating prolonged disease control. The SOLO-1 trial estimated a median PFS of 56.0 months [17]. In SOLO-1, median OS was not reached but a 7-year survival rate was reported at 67% [17]. Our data demonstrated a median OS of 59 months, likely influenced by the shorter follow-up time compared to SOLO-1 (median follow-up was 88.9 months) [18]. The average age of patients in our study was 63 years, compared with 54 years in SOLO-1 [19]. We show here that BRCA2 germline-mutated patients have better PFS compared to BRCA1 which is comparable to SOLO-1 where BRCA2 carriers had a larger drop in hazard ratios (HR 0.20, 80% risk reduction) than BRCA1 carriers (HR 0.41, 59% risk reduction) [20]. Our data supports pre-clinical studies which show that BRCA2-deficient cells have a 133-fold PARPi sensitivity compared to 57-fold PARPi sensitivity in BRCA1 models [21]. This biological vulnerability may reflect BRCA2’s critical role in RAD51 loading during homologous recombination repair [22]. In contrast to our findings, a meta-analysis of 11 randomized controlled trials (RCTs) reported comparable PARPi efficacy (pooled PFS HR:0.42 [95% CI: 0.35–0.50] for BRCA1 vs. 0.35 [95% CI: 0.24–0.51] for BRCA2 [23]. However, the latter study was limited by diverse groups of patients receiving various PARPi [23]. Interestingly, a recently published multi-centre real world study investigated the benefit of olaparib in 140 tumours based on BRCA1/2 mutation subtypes. The authors reported significant survival benefit in BRCA2-mutated patients with RAD51-binding domain mutations, and in BRCA1 patients with mutations in RING and BRCT, domains compared to other mutation subtypes [24]. These data suggest a complex role for individual BRCA1/2 mutations in influencing the benefit of olaparib. Future trials of next-generation PARP1-specific inhibitors should include this subgroup analysis to clarify the data further.

In BRCA–wild-type patients, primary niraparib therapy led to a median PFS of 11 months which is slightly less than that reported in the PRIMA trial (13.8 months) [25]. As BRCA1/2 mutated and HRD patients also received niraparib in the PRIMA study, this likely contributed to the better PFS observed in that study [25]. We reported a PFS of 10 months for non-BRCA-mutated patients who received niraparib in the recurrent disease setting. The ENGOT-OV16/NOVA trial showed a similar PFS of 9.3 months in patients without a germline mutation in BRCA [26]. In the recurrent disease setting, no significant PFS or OS difference was observed between niraparib (median PFS 10.0 months) and rucaparib (median PFS 9.0 months; *p* = 0.594), supporting toxicity-guided agent selection, especially in light of niraparib’s higher hematological toxicity risk [27]. A significant limitation to our real-world study is that we had HRD data for only 22% of our patients, largely due to only recent availability of HRD testing within the NHS. Despite this limitation, we observed numerically better PFS in HRD compared to HRP and the data was comparable to that reported in the PRIMA [25] and PAOLA-1 trials [28]. However, a larger cohort of patients where HRD status is available should be evaluated to confirm the real-world evidence shown here.

In our cohort, the majority of patients received interval debulking surgery post neoadjuvant chemotherapy (62/80), and only 14/80 patients received upfront surgery. We observed a numerically better but non-significant PFS of 37 months in the upfront surgery group compared to the interval debulking surgery group (19 months). Similarly, median OS was also better but non-significant in the upfront surgery group (53 months) compared to the interval debulking surgery group (40 months). We cannot confirm causality as statistical significance was not reached in our study. However, in the recently reported TRUST trial [29], upfront surgery showed significantly improved median PFS (22.2 months) and showed a trend toward longer overall survival (OS) (54 months), compared to neoadjuvant chemotherapy, followed by interval cytoreductive surgery (19.7 months and 48.3 months, respectively. All patients in this retrospective study received PARP inhibitors. In all, 66/80 received a PARPi as first-line maintenance and 14/80 had PARPi maintenance in the recurrent setting. In the primary maintenance setting 21/66 received olaparib, 43/66 received niraparib, and 2/66 received rucaparib. In the recurrent setting 1/14 received olaparib, 3/14 received niraparib, and 10/14 received rucaparib.

The real-world data presented here is from a large NHS (National Health Service, UK) tertiary care cancer centre in England. Our data are consistent with recent RWEs from Italian [30], Chinese [31], American [32,33], Spanish [34], French [35], and Japanese [36] cohorts. Moreover, the data presented here specifically provides valuable insights from an NHS context and could inform real-world clinical decision-making in the UK. Limitations to our study include many subgroup comparisons, retrospective nature, incomplete HRD data, single centre bias, and short follow-up. We therefore caution against overinterpretation of the data.

Clinical trial evidence and the RWE data, including ours, have shown that PARP inhibitor (PARPi) maintenance therapy can significantly prolong survival in patients with HGSOCs, but the therapeutic benefit is not sustained. Intrinsic or acquired secondary resistance to PARPi therapy is an emerging clinical challenge [6,7,37,38,39]. Reversion of the BRCA1/2 mutation can lead to clinical PARPi resistance in BRCA–germline-mutated ovarian cancer. However, in the more common platinum-sensitive sporadic HGSOC, the clinical mechanisms of development of PARPi resistance are complex and yet to be fully defined. Novel strategies in PARPi resistant HGSOCs are an area of intense clinical investigation. Alternative therapeutic strategies such as those targeting ATM, ATR, WEE1, Polθ, CDK 4/6, CDK12, glucocorticoid receptor modulators, antibody drug conjugates (ADCs), and immune checkpoint inhibition hold promise in PARPi resistant HGSOCs [6,7,37,38,39].

## 5. Conclusions

In summary, our study reaffirms PARPi efficacy across diverse real-world populations. Real-world evaluation in BRCA1/2 germline-deficient populations with a focus on differential PARPi efficacy and primary versus interval debulking surgery will help optimize the management of patients with HGSOCs.

## Figures and Tables

**Table 1:** Patient clinical characteristics and PARPi treatment, BRCA status and treatment settings.

**Mean age in years** **(min, max)**
63.06 (28, 88)
**Duration of maintenance Rx in months, mean (min, max)**
13.74 (0, 63)
**Median follow-up time (months)**
28 (months)
**BRCA mutation, No. (%)**
Wild type 131 (74%)
BRCA1 + 25 (14.1%)
BRCA2 + 21 (11.9%)
**HRD, No. (%)**
HR-deficient 20 (11.3%)
HRD proficient 19 (10.7%)
Not available 138 (78%)
**PARPI No. (%)**
Olaparib 37 (20.9%)
Niraparib 95 (53.7%)
Rucaparib 38 (21.5%)
Olaparib + Bevacizumab 7 (4%)
**PARPI Rx setting, No. (%)**
Primary maintenance 94 (53.1%)
Recurrence 83 (46.9%)
**BMI, No. (%)**
< 25, 61 (34.5%)
≥ 25, 116 (65.5%)
**Surgical intervention, No. (%)**
Upfront surgery 14 (17.5%)
Interval debulking surgery 62 (77.5%)
Inoperable 4 (5%)
**FIGO stage, No. (%)**
I, 4/177 (2.25%)
II, 2/177 (1.12%)
III, 101/177 (57.06%)
IV, 70/177 (39.5%)
**ECOG performance status, No. (%)**
0, 58 (32.8%)
1, 107 (60.4%)
2, 12 (6.8%)
**Co-morbidities, No. (%)**
HTN, 41 (23.1%)
Type II Diabetes Mellitus, 10 (5.6%)
IHD, 3 (1.69%)
Hypothyroidism, 9 (5.06%)
Asthma/COPD, 15 (8.4%)
No comorbidities, 81 (45.7%)
**Radiological response post chemotherapy (prior to PARPi initiation), No. (%)**
Complete response, 39, (22%)
Partial response, 110, (62.1%)
Stable disease, 9, (5.08%)
Mixed response, 4 (2.25%)
No radiology imaging available, 15 (8.4%)
**Chemotherapy regimen prior to PARPI, No. (%)**
Platinum doublet chemotherapy, 144 (81.3%)
Platinum doublet + Bevacizumab, 2 (1.12%)
Platinum single agent, 31 (17.5%)

**Table 2:** Summary of survival outcomes by subgroups.

Subgroup	n	Median PFS (Months)	*p*-Value	Median OS (Months)	*p*-Value
BRCA1 vs. BRCA2 (olaparib, primary)	10 vs. 12	29.0 vs. NR	0.002	49.0 vs. NR	0.163
BRCA–wild-type (niraparib, primary)	60	11.0	–	29.0	–
Upfront vs. interval surgery	14 vs. 62	37.0 vs. 19.0	0.49	53.0 vs. 40.0	0.50
Niraparib vs. rucaparib (recurrent)	35 vs. 34	10.0 vs. 9.0	0.59	–	–
HRD vs. HRP (primary)	20 vs. 19	21.0 vs. NR	0.71	–	–

## Data Availability

Raw data will be made available upon reasonable request.

## Abbreviations

The following abbreviations are used in this manuscript:

HGSOC	High-grade serous ovarian cancers
HRD	Homologous recombination deficiency
HRP	Homologous recombination proficiency
PFS	Progression free survival
OS	Overall Survival
PARP	Poly-ADP ribose polymerase
GMSA	Genomic Medicine Service Alliance
NHS	National Health Service

## References

[1] (2024). CRUK. https://www.cancerresearchuk.org/about-cancer/ovarian-cancer.

[2] Lheureux S., Braunstein M., Oza A.M. (2019). Epithelial ovarian cancer: Evolution of management in the era of precision medicine. CA Cancer J. Clin..

[3] Piver M.S. (2006). Treatment of ovarian cancer at the crossroads: 50 years after single-agent melphalan chemotherapy. Oncology.

[4] Drew Y. (2015). The development of PARP inhibitors in ovarian cancer: From bench to bedside. Br. J. Cancer.

[5] Chen S., Parmigiani G. (2007). Meta-analysis of BRCA1 and BRCA2 penetrance. J. Clin. Oncol..

[6] Kulkarni S., Gajjar K., Madhusudan S. (2024). Poly (ADP-ribose) polymerase inhibitor therapy and mechanisms of resistance in epithelial ovarian cancer. Front. Oncol..

[7] Kulkarni S., Seneviratne N., Tosun C., Madhusudan S. (2025). PARP inhibitors in ovarian cancer: Mechanisms of resistance and implications to therapy. DNA Repair.

[8] Krishnakumar R., Kraus W.L. (2010). The PARP side of the nucleus: Molecular actions, physiological outcomes, and clinical targets. Mol. Cell.

[9] Kulkarni S., Brownlie J., Jeyapalan J.N., Mongan N.P., Rakha E.A., Madhusudan S. (2022). Evolving DNA repair synthetic lethality targets in cancer. Biosci. Rep..

[10] Mateo J., Lord C.J., Serra V., Tutt A., Balmana J., Castroviejo-Bermejo M., Cruz C., Oaknin A., Kaye S.B., de Bono J.S. (2019). A decade of clinical development of PARP inhibitors in perspective. Ann. Oncol..

[11] Pilie P.G., Tang C., Mills G.B., Yap T.A. (2019). State-of-the-art strategies for targeting the DNA damage response in cancer. Nat. Rev. Clin. Oncol..

[12] Murai J., Huang S.Y., Das B.B., Renaud A., Zhang Y., Doroshow J.H., Ji J., Takeda S., Pommier Y. (2012). Trapping of PARP1 and PARP2 by Clinical PARP Inhibitors. Cancer Res..

[13] George A., Kaye S., Banerjee S. (2017). Delivering widespread BRCA testing and PARP inhibition to patients with ovarian cancer. Nat. Rev. Clin. Oncol..

[14] National Institute for Health and Care Excellence Olaparib for Maintenance Treatment of BRCA Mutation-Positive Advanced Ovarian, Fallopian Tube or Peritoneal Cancer After Response to FIRST-Line Platinum-Based Chemotherapy. [TA962]. https://www.nice.org.uk/guidance/ta962.

[15] National Institute for Health and Care Excellence Niraparib for Maintenance Treatment of Advanced Ovarian, Fallopian Tube and Peritoneal Cancer After Response to First-Line Platinum-Based Chemotherapy. [TA673]. https://www.nice.org.uk/guidance/ta673.

[16] National Institute for Health and Care Excellence Rucaparib for Maintenance Treatment of Advanced Ovarian, Fallopian Tube and Peritoneal Cancer After Response to First-Line Platinum-Based Chemotherapy. [TA1055]. https://www.nice.org.uk/guidance/ta1055.

[17] Banerjee S., Moore K.N., Colombo N., Scambia G., Kim B.G., Oaknin A., Friedlander M., Lisyanskaya A., Floquet A., Leary A. (2021). Maintenance olaparib for patients with newly diagnosed advanced ovarian cancer and a BRCA mutation (SOLO1/GOG 3004): 5-year follow-up of a randomised, double-blind, placebo-controlled, phase 3 trial. Lancet Oncol..

[18] DiSilvestro P., Banerjee S., Colombo N., Scambia G., Kim B.G., Oaknin A., Friedlander M., Lisyanskaya A., Floquet A., Leary A. (2023). Overall Survival With Maintenance Olaparib at a 7-Year Follow-Up in Patients With Newly Diagnosed Advanced Ovarian Cancer and a BRCA Mutation: The SOLO1/GOG 3004 Trial. J. Clin. Oncol..

[19] Moore K., Colombo N., Scambia G., Kim B.G., Oaknin A., Friedlander M., Lisyanskaya A., Floquet A., Leary A., Sonke G.S. (2018). Maintenance Olaparib in Patients with Newly Diagnosed Advanced Ovarian Cancer. N. Engl. J. Med..

[20] DiSilvestro P., Colombo N., Scambia G., Kim B.G., Oaknin A., Friedlander M., Lisyanskaya A., Floquet A., Leary A., Sonke G.S. (2020). Efficacy of Maintenance Olaparib for Patients With Newly Diagnosed Advanced Ovarian Cancer With a BRCA Mutation: Subgroup Analysis Findings From the SOLO1 Trial. J. Clin. Oncol..

[21] Farmer H., McCabe N., Lord C.J., Tutt A.N., Johnson D.A., Richardson T.B., Santarosa M., Dillon K.J., Hickson I., Knights C. (2005). Targeting the DNA repair defect in BRCA mutant cells as a therapeutic strategy. Nature.

[22] Holloman W.K. (2011). Unraveling the mechanism of BRCA2 in homologous recombination. Nat. Struct. Mol. Biol..

[23] Li S., Tao L., Dai H., Gong X., Zhuo Y., Xiang H., Zhao Y., Gao Q., Deng L. (2021). BRCA1 Versus BRCA2 and PARP Inhibitors Efficacy in Solid Tumors:A Meta-Analysis of Randomized Controlled Trials. Front. Oncol..

[24] Marchetti C., Fagotti A., Fruscio R., Cassani C., Incorvaia L., Perri M.T., Sassu C.M., Camnasio C.A., Giudice E., Minucci A. (2025). Benefit from maintenance with PARP inhibitor in newly diagnosed ovarian cancer according to BRCA1/2 mutation type and site: A multicenter real-world study. ESMO Open.

[25] Gonzalez-Martin A., Pothuri B., Vergote I., DePont Christensen R., Graybill W., Mirza M.R., McCormick C., Lorusso D., Hoskins P., Freyer G. (2019). Niraparib in Patients with Newly Diagnosed Advanced Ovarian Cancer. N. Engl. J. Med..

[26] Mirza M.R., Monk B.J., Herrstedt J., Oza A.M., Mahner S., Redondo A., Fabbro M., Ledermann J.A., Lorusso D., Vergote I. (2016). Niraparib Maintenance Therapy in Platinum-Sensitive, Recurrent Ovarian Cancer. N. Engl. J. Med..

[27] Xu Y., Ding L., Tian Y., Bi M., Han N., Wang L. (2020). Comparative Efficacy and Safety of PARP Inhibitors as Maintenance Therapy in Platinum Sensitive Recurrent Ovarian Cancer: A Network Meta-Analysis. Front. Oncol..

[28] Ray-Coquard I., Pautier P., Pignata S., Perol D., Gonzalez-Martin A., Berger R., Fujiwara K., Vergote I., Colombo N., Maenpaa J. (2019). Olaparib plus Bevacizumab as First-Line Maintenance in Ovarian Cancer. N. Engl. J. Med..

[29] Mahner S.H.F., Salehi S., Reuss A., Guyon F., Du Bois A., Harter P., Fotopoulou C., Querleu D., Mosgaard B.J. (2025). TRUST: Trial of radical upfront surgical therapy in advanced ovarian cancer (ENGOT-ov33/AGO-OVAR OP7). J. Clin. Oncol..

[30] Loverro M., Marchetti C., Salutari V., Giannarelli D., Vertechy L., Capomacchia F.M., Caricato C., Campitelli M., Panico C., Avesani G. (2025). Real-world outcomes of PARP inhibitor maintenance in advanced ovarian cancer: A focus on disease patterns and treatment modalities at recurrence. ESMO Open.

[31] Zhao M., Qiu S., Wu X., Miao P., Jiang Z., Zhu T., Xu X., Zhu Y., Zhang B., Yuan D. (2023). Efficacy and Safety of Niraparib as First-Line Maintenance Treatment for Patients with Advanced Ovarian Cancer: Real-World Data from a Multicenter Study in China. Target. Oncol..

[32] Pan Y.E., Hood A., Ahmad H., Altwerger G. (2023). Real-World Efficacy and Safety of PARP Inhibitors in Recurrent Ovarian Cancer Patients With Somatic BRCA and Other Homologous Recombination Gene Mutations. Ann. Pharmacother..

[33] Richardson D.L., Quintanilha J.C.F., Danziger N., Li G., Sokol E., Schrock A.B., Ebot E., Bhardwaj N., Norris T., Afghahi A. (2024). Effectiveness of PARP Inhibitor Maintenance Therapy in Ovarian Cancer by BRCA1/2 and a Scar-Based HRD Signature in Real-World Practice. Clin. Cancer Res..

[34] Tuninetti V., Marin-Jimenez J.A., Valabrega G., Ghisoni E. (2024). Long-term outcomes of PARP inhibitors in ovarian cancer: Survival, adverse events, and post-progression insights. ESMO Open.

[35] Rippstein N., Zemmour C., Rodrigues M., Ray-Coquard I., Gladieff L., Pautier P., Frenel J.S., Costaz H., Lebreton C., Pomel C. (2025). PARP inhibitors as maintenance therapy in ovarian cancer after platinum-sensitive recurrence: Real-world experience from the Unicancer network. Oncologist.

[36] Uekusa R., Yokoi A., Watanabe E., Yoshida K., Yoshihara M., Tamauchi S., Shimizu Y., Ikeda Y., Yoshikawa N., Niimi K. (2024). Safety assessments and clinical features of PARP inhibitors from real-world data of Japanese patients with ovarian cancer. Sci. Rep..

[37] Roufai D.B., Toth R., Palo U., Scaglione G., Palacios A.T., Novak Z., El Hajj H., Shushkevich A., Ledermann J. (2025). Mechanisms of drug resistance: PARP inhibitors, antibody-drug-conjugates, and immunotherapy. Int. J. Gynecol. Cancer.

[38] Wang Z., Liu Y., Yang Q. (2025). Navigating PARP Inhibitor Resistance in Ovarian Cancer: Bridging Mechanistic Insights To Clinical Translation. Curr. Treat. Options Oncol..

[39] Zou Y., Zhang H., Chen P., Tang J., Yang S., Nicot C., Guan Z., Li X., Tang H. (2025). Clinical approaches to overcome PARP inhibitor resistance. Mol. Cancer.

